# Inconsistent Effect of Arousal on Early Auditory Perception

**DOI:** 10.3389/fpsyg.2017.00447

**Published:** 2017-03-29

**Authors:** Anna C. Bolders, Guido P. H. Band, Pieter Jan M. Stallen

**Affiliations:** ^1^Cognitive Psychology Unit, Institute of Psychology, Leiden UniversityLeiden, Netherlands; ^2^Leiden Institute for Brain and Cognition, Leiden UniversityLeiden, Netherlands

**Keywords:** mood, arousal, hearing, masked-auditory threshold, psychophysics, affective modulation, auditory perception

## Abstract

Mood has been shown to influence cognitive performance. However, little is known about the influence of mood on sensory processing, specifically in the auditory domain. With the current study, we sought to investigate how auditory processing of neutral sounds is affected by the mood state of the listener. This was tested in two experiments by measuring masked-auditory detection thresholds before and after a standard mood-induction procedure. In the first experiment (*N* = 76), mood was induced by imagining a mood-appropriate event combined with listening to mood inducing music. In the second experiment (*N* = 80), imagining was combined with affective picture viewing to exclude any possibility of confounding the results by acoustic properties of the music. In both experiments, the thresholds were determined by means of an adaptive staircase tracking method in a two-interval forced-choice task. Masked detection thresholds were compared between participants in four different moods (calm, happy, sad, and anxious), which enabled differentiation of mood effects along the dimensions arousal and pleasure. Results of the two experiments were analyzed both in separate analyses and in a combined analysis. The first experiment showed that, while there was no impact of pleasure level on the masked threshold, lower arousal was associated with lower threshold (higher masked sensitivity). However, as indicated by an interaction effect between experiment and arousal, arousal did have a different effect on the threshold in Experiment 2. Experiment 2 showed a trend of arousal in opposite direction. These results show that the effect of arousal on auditory-masked sensitivity may depend on the modality of the mood-inducing stimuli. As clear conclusions regarding the genuineness of the arousal effect on the masked threshold cannot be drawn, suggestions for further research that could clarify this issue are provided.

## Introduction

Affective states, such as moods and emotions are thought to facilitate adaptive responding to situational demands. Several studies have demonstrated that changes in emotional states are associated with changes in perceptual or cognitive processes, including visual perception (Gasper, 2004; Phelps et al., 2006; Bocanegra and Zeelenberg, 2009; Kuhbandner et al., 2009; Lee et al., 2014a), temporal attention (Jefferies et al., 2008), spatial attention (Phelps et al., 2006), and cognitive control (van Wouwe et al., 2009; van Steenbergen et al., 2010; Kuhbandner and Zehetleitner, 2011). Influences of mood on basic auditory processing, however, have remained largely unexplored [see Siegel and Stefanucci (2011) for a recent exception]. To fill this gap, we investigated the effects of mood state on the masked-auditory threshold in two experiments. In the first experiment, mood was induced by imagining a mood-appropriate event while listening to mood inducing music. In the second experiment, we used a visual mood induction procedure.

Although there has been little research interest in mood-induced modulation of auditory compared to visual processing, mood-induced modulation in the auditory domain does seem highly plausible. First, it has been argued that the auditory system is particularly suitable to function as alarm system because the auditory system enables detection of potentially relevant stimuli, within as well as outside of our visual field of view (Juslin and Västfjäll, 2008; Asutay and Västfjäll, 2015). Modulation of the auditory system by affective state may enhance detection of these potentially relevant stimuli and thereby increase chances of survival. For example, the need for an organism to invest in high auditory sensitivity may be higher in dangerous conditions, associated with more negative or aroused affective states, than in safe conditions, associated with more positive or relaxed affective states. Second, evidence is accumulating that the auditory system is well equipped to adapt to demands of the environment (Fritz et al., 2007; Robinson and McAlpine, 2009). Animal studies demonstrate that gain control mechanisms are operating at multiple levels in the auditory system (Robinson and McAlpine, 2009), for example in the inferior colliculus (Dean et al., 2005) in the midbrain and the auditory cortex (Rabinowitz et al., 2011). In addition, neuronal receptive fields can reshape rapidly as a consequence of changes in task demands (Fritz et al., 2007) and descending pathways modulate neuronal responses to signals in noise in the auditory nerve (Kawase et al., 1993), the cochlear nucleus (Mulders et al., 2008), and the inferior colliculus (Seluakumaran et al., 2008). These forms of plasticity in the auditory system enable enhanced coding of salient or relevant stimuli (Fritz et al., 2007; Robinson and McAlpine, 2009) and may enable affective modulation. Furthermore, Fritz et al. (2007) conjecture that the rapid adaptations in tuning in the auditory cortex are mediated by neuromodulators such as dopamine (DA), norepinephrine (NE), and serotonin (5HT). Activity changes of these neuromodulators are also implicated in changes in affective state (NE, Aston-Jones et al., 1996; DA, Ashby et al., 1999; 5HT, Mitchell and Phillips, 2007), which may hint at a neural mechanism for affective modulation of the auditory system. Taken together, mood modulation of auditory processing appears plausible on functional as well as on neural grounds.

While studies of mood effects on audition are rare, several effects of brief affective states on auditory processing have been reported. For example, the wave V of the brain stem auditory-evoked potentials (BAEPs), an early reflection of inferior colliculus activity in the brainstem, was modulated by fear of mild electric shock (Baas et al., 2006). This suggests that heightened activation of structures involved in defensive states, such as the amygdala and locus coeruleus (LC), modulate auditory processing in the brainstem. In addition, using a similar threat-of-shock paradigm, Al-Abduljawad et al. (2008) showed that also a later component of the auditory-evoked potential, the N1/P2, was potentiated in threatening conditions.

In contrast to the two above-mentioned studies that involved brief affect inductions, in the current study we investigated modulation of auditory processing by mood, which is a more diffuse affective state longer in duration (Gray and Watson, 2007). In addition, following previous studies that demonstrated effects of emotion cues on contrast sensitivity in the visual domain (Phelps et al., 2006; Bocanegra and Zeelenberg, 2009; Lee et al., 2014a), we used a perceptual performance measure rather than brain indices of auditory processing. To the best of our knowledge only one other recent study has investigated how mood impacts basic auditory perception (Siegel and Stefanucci, 2011). In this study, a negative mood was induced by means of an autobiographical memory writing task after which participants were asked to rate duration and loudness of short neutral tones on an anchored scale. Sounds were judged as louder by participants in an anxious mood compared to participants in a neutral mood. No differences were found in duration perception between the two groups. These findings not only provide further evidence for affective modulation of auditory processing, but also raise several questions that we aim to answer in the current study.

A first question that arises is whether the mood effects on loudness judgment observed by Siegel and Stefanucci (2011) might actually reflect response bias, rather than modulation of perceptual sensitivity (Odgaard et al., 2003; Marks and Florentine, 2011). Response bias is determined by the (implicit) criterion, or rule, an observer employs in translating sensory information into overt responses. Measures of magnitude on a subjective scale are assumed to be prone to effects of response bias (Dalton, 1996; Odgaard et al., 2003). In order to rule out that alternative explanation, in the present study, we used a performance measure of auditory perception that minimizes such biases. A two-interval-forced choice (2IFC) procedure was combined with a staircase procedure (García-Pérez, 1998) to measure the masked-auditory detection threshold for pure tones, which reflects listeners’ ability to detect faint sounds in noise. In terms of signal detection theory, the 2IFC procedure is regarded a criterion-free measure, i.e., it measures sensitivity irrespective of the response criterion used by the observer (Green and Swets, 1966; Odgaard et al., 2003; Gillmeister and Eimer, 2007; Kingdom and Prins, 2010).

We chose to measure the masked-auditory threshold (in noise) rather that the absolute threshold of hearing (in quiet) for two reasons. First, in real life listening our ability to detect faint sounds is almost always limited by the ambient noise that masks those sounds and not by our absolute sensitivity to those sounds (cf. Moore, 2012). Second, the adaptive 2IFC procedure measuring the masked-auditory threshold was shown to have better reliability (lower intrasubject standard deviation) than the same procedure measuring the absolute threshold (Marshall et al., 1996). To emphasize that the masked-auditory threshold not only depends on sensitivity to the detected faint tones, but also on the effects of the masking noise, we will refer to the inverse of the masked threshold as masked sensitivity.

The masked-auditory threshold task in the current study involved detecting a 1-kHz tone signal in a constant white noise as masker. These masking conditions are often labeled as simultaneous masking and energetic masking conditions (Moore, 2012). Simultaneous masking refers to the situation where the tone and mask are presented simultaneously. Energetic masking refers to the situation where masking results from overlap in the excitation patterns of the signal and of the noise at the level of the auditory periphery (Oxenham et al., 2003; Moore, 2012). Simultaneous energetic masking can be largely explained by frequency tuning of the basilar membrane in the cochlea (Moore, 2012; Recio-Spinoso and Cooper, 2013). This does not, however, exclude the possibility of (top–down) modulation affecting the masked-auditory threshold. Even cochlear responses are thought to be susceptible to modulation through efferent pathways from higher centers of brain to the outer hair cells (Smith et al., 2012), which may explain effects found of cueing and expectancy on masked sensitivity (Tan et al., 2008). Furthermore, as described above, higher auditory centers can further modulate the signal coming from the cochlea by gain control mechanisms and through rapid reshaping of neuronal receptive fields.

A second question that arises from the findings of Siegel and Stefanucci (2011) concerns which aspect of the affective state contributed to the modulation. According to emotion theorists affective states can be described by two main dimensions, pleasure (or valence) and arousal (or activation, Yik et al., 1999; Russell, 2003). Pleasure reflects the hedonic value of the affective state, ranging from unpleasant to pleasant, and arousal reflects the sensation of activation or energy mobilization, ranging from sleepy to activated (Russell, 2003). Previous studies have demonstrated specific pleasure or arousal effects depending on the type of cognitive abilities measured (Jefferies et al., 2008; van Steenbergen et al., 2010; Kuhbandner and Zehetleitner, 2011). Furthermore, different neuromodulatory systems may mediate different affective states. Hedonic value is often associated with DA (Ashby et al., 1999), while arousal is associated with NE (Aston-Jones et al., 1996). Neuro-computational models relate both DA and NE activity to gain modulation at the neuronal level, which at the functional level changes the ability to detect a signal from a noise background (Servan-Schreiber et al., 1990). As described above, both neuromodulators may play a role in situational adaptation of the auditory system.

Siegel and Stefanucci (2011) compared loudness perception only between participants in an anxious and a neutral state. An anxious state is both lower in pleasure level and higher in arousal level than a neutral state. It thus remains to be answered whether affective modulation of loudness depends on pleasure or arousal, or a combination of both. To disentangle the effects of pleasure and arousal on auditory perception, in the present study we used a standard mood induction procedure to elicit four different moods that can be differentiated along the dimensions arousal and pleasure (Jefferies et al., 2008; van Steenbergen et al., 2010; Kuhbandner and Zehetleitner, 2011). Moods were induced in four different groups of participants: anxious (low pleasure and high arousal), sad (low pleasure and low arousal), happy (high pleasure and high arousal), and calm (high pleasure and low arousal). Comparison of auditory perception between these groups allowed assessing the separate contribution of pleasure and arousal.

Theoretical accounts of effects of the pleasure dimension of mood on perception and cognition (e.g., Derryberry and Tucker, 1994; Fredrickson, 2004) have not explicitly dealt with basic auditory information processing. Therefore, predictions for the present study based on these theories can only be formulated indirectly. With respect to basic visual perception a widely accepted claim is that positive mood broadens the perceptual scope, while negative mood narrows it (Derryberry and Tucker, 1994; Gasper and Clore, 2002; Fredrickson and Branigan, 2005). Experiments using a visual global–local task have confirmed that people in a positive mood attend more to global features of a figure and less to the smaller details than people in a negative mood (Gasper and Clore, 2002; Fredrickson and Branigan, 2005). Furthermore, stronger interference of irrelevant stimuli flanking a target stimulus in positive than in negative mood also suggests that the scope of spatial attention is broadened in positive mood (Rowe et al., 2007). There is no one-to-one correspondence between global and local visual processing tasks, tasks measuring spatial breadth of visual attention, and the masked-auditory threshold task. However, it has been suggested that frequency in the auditory domain may play a similar role in attentional selectivity as spatial location does in the visual domain (Woods et al., 2001). Therefore, increased breadth of attentional scope in the visual domain may be reflected in decreased frequency selectivity in the auditory domain, thereby increasing the threshold for a specific frequency in a noise background. If positive mood broadens frequency tuning, while negative mood leads to more narrow tuning, thresholds are expected to be lower in negative than in positive moods.

The above-mentioned theories on mood and perceptual scope do not accommodate effects of affective arousal regardless of pleasure on perception and cognition. A classic observation is the inverted U-shaped relation between arousal and performance on various (perceptual and perceptual-motor) tasks (Yerkes and Dodson, 1908; Easterbrook, 1959; Kahneman, 1973; Anderson, 1990; Aston-Jones and Cohen, 2005). If arousal similarly influences auditory perceptual performance, it is expected that there is an optimal level of arousal at which masked-auditory thresholds will be lowest; at levels below and above this optimum, thresholds will be higher.

## Experiment 1: Masked-Auditory Threshold in Moods Induced by Music and Imagining

### Method

#### Participants

Eighty-one participants (Age: *M* = 20.5, *SD* = 2.0, and 18–27 years; 20 males) with no self-reported hearing problems or depression took part either for course credit or payment (€5). They were randomly assigned to one of four mood groups: calm, happy, sad, and anxious. Data from five participants were not included in the analyses because they had strongly deviating baseline or test thresholds (above the three inter-quartile range criterion of their assigned mood group).

#### Apparatus

Stimulus presentation was controlled by E-prime 2 (Schneider et al., 2002) using a computer with a CRT screen (75 Hz refresh rate, 1024 × 768 resolution). Responses were made on a QWERTY keyboard and by using a mouse. Sound was binaurally presented through insert earphones (Etymotic ER-4B microPro) with 3-flange eartips that provide 35 dB external noise attenuation.

#### Sound Levels

Sound levels at output were calculated from the voltages delivered at the earphone input as measured with an oscilloscope (Type Tektronix TDS2002) and the earphone efficiency as provided by the earphone manufacturer (108 dB SPL for 1 Vrms in a Zwislocki coupler, ER-4 datasheet, Etymotic Research, 1992).

#### Mood Induction and Assessment

Mood was induced by listening to music and imagining a mood-appropriate event. This standard procedure has been shown to elicit reliable changes in mood (Eich et al., 2007) and has been successfully used in previous studies (Jefferies et al., 2008; van Steenbergen et al., 2010; Kuhbandner and Zehetleitner, 2011). Following these examples we manipulated mood according to two factors (pleasure and arousal). No neutral control condition was included. The power of such a design is larger than when all mood conditions need to be compared to a neutral condition. Furthermore, it is rather difficult to establish a neutral mood condition. This becomes apparent from the results of Jefferies et al. (2008), who initially included a neutral (no induction procedure) condition, but assigned these participants later to different mood groups on the basis of their subjective arousal and pleasure ratings.

Participants were instructed to get into the desired mood by vividly imagining and writing down in detail a mood-appropriate event, either based on their own past experience or on a given scenario. Simultaneously, they listened to a selection of classical music excerpts which were validated to promote a particular mood (Jefferies et al., 2008). An overview of the scenarios and musical pieces used per mood condition can be found in **Table 1**. Per condition two excerpts were combined to one mp3 file with a minimum duration of 11 min to cover the duration of the mood induction procedure. The root mean square (RMS) value of each excerpt was first normalized to the average RMS value of the excerpts using RMS-based normalization with equal loudness contours in Cool Edit pro software. Further, the combined files were normalized across conditions to the same level also using RMS-based normalization with the equal loudness contour in Cool Edit pro.

**Table 1:** Music and example scenarios per mood condition as used in the mood induction procedure of Experiment 1.

Mood condition	Name (and composer) of musical piece	Duration of musical piece (min:sec)^a^	Example scenario (text translated from Dutch and slightly shortened)
Anxious (low pleasure and high arousal)	Mars, The Bringer of War (Holst) Uranus the Magician (Holst)	07:12 06:05	Together with a good friend you are taking a roller coaster ride in an amusement park. At the moment you drive off you realize that the safety lever is lose. You are in danger of falling out!

Sad (Low pleasure Low arousal)	Piano Quintet No. 1 in D Minor (Fauré) Violin Concerto: Adagio di Molto (Sibelius)	08:11 08:06	You are visiting a good friend who is ill in bed. You are told the bad news that your friend is very seriously ill and does not have much longer to live. This will likely be the last time that you will see your friend.

Happy (high pleasure and high arousal)	Eine Kleine Nachtmusik: Allegro (Mozart) The Nutcracker: Waltz of the Flowers (Tchaikovsky)	06:31 06:23	You are in a shop together with a friend. On a whim, you decide to buy a scratch card. After scratching the card you find out that you’ve won the jackpot of 50.000 euro!

Calm (high pleasure and low arousal)	Venus, The Bringer of Peace (Holst) Ave Maria (Bach)	09:33 04:23	You arrive home after a long day at work. You take a well-deserved warm bath to let your tired body rest. The foam and warmth of the water make you dream away about faraway places.

*^a^Music stopped playing when the mood induction procedure was finished.*

Equivalent continuous sound level (*L*_eq_), for each music file was estimated using the following procedure: RMS values per 4.5 ms time-window of the first 10 min and 20 s (the average duration of the mood induction procedure) of each music file were computed. Next, voltages at earphone input were estimated for each window from the ratio of the RMS amplitude and the measured voltage at earphone input for a 1-kHz tone. Subsequently, *L*_eq_ at output was computed from the estimated voltages at earphone input and earphone efficiency. The estimated level, for all files, was approximately *L*_eq_ = 49.5 (±1) dB. Note that this level was kept well below *L*_eq_ = 70 dBA to avoid effects of music exposure on the masked-auditory threshold. Previous research has shown that after 10 min of loud noise (e.g., 105 dB SPL), a temporary shift in auditory thresholds occurs, while after exposure to low level music (*L*_eq_ = 70 dBA) the auditory threshold is not altered (Miyakita et al., 1992).

Over the course of the experiment, participants rated their current mood six times on an electronic version of the 9 × 9 affect grid (Russell, 1989). Pleasure was indicated on the horizontal axis [extremely unpleasant (1) to extremely pleasant (9)] and arousal on the vertical axis [extremely low arousal (1) to extremely high arousal (9)]. These ratings were used to check if the induction procedure had succeeded.

#### Threshold Task

##### Sounds

For all sounds used in the threshold task, digital sound properties were standardized (44 kHz, 16 bit, mono and binaural). The signal was a 500-ms, 1-kHz pure tone with 10 ms ramped on- and offset, presented at a sound level of 68 dB SPL as initial value for the adaptive procedure. An empty sound file of 500 ms served as non-signal. Both files were created with Audacity software. The masking noise that was constantly present during the threshold tasks was white noise (20 Hz–10 kHz band-filtered) generated in Goldwave software. The white noise was presented with a voltage delivered at the earphone input that would equal 38 dB SPL output for 1 kHz tone (108 dB SPL/1 Vrms).

##### Task procedure

**FIGURE 1:**
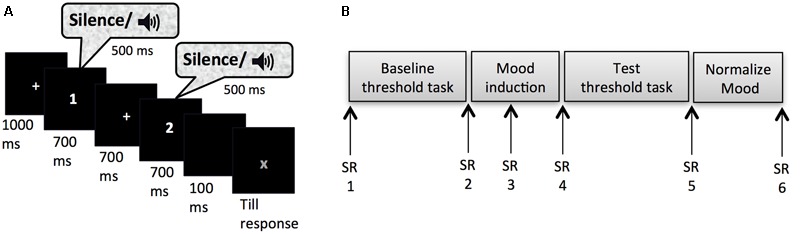
**(A)** Trial set-up of the threshold task. **(B)** Experimental timeline for Experiment 1 and 2; SR, subjective rating of pleasure and arousal on the affect grid.

Masked-auditory thresholds were determined twice (pre- and post-mood induction) by means of an adaptive 2IFC task. **Figure 1A** shows the trial structure of this task. Each trial started with a fixation cross presented in the center of the screen for 1000 ms. This was followed by two observation-intervals each of 700 ms indicated with a number presented in the center of the screen (1 or 2) and separated by an inter-observation interval of 700 ms. On each trial one of the two observation intervals was randomly selected to contain the signal with the constraint that maximally four trials with the same selected interval could occur in succession. The 500 ms signal was centered in the observation interval. The second observation interval was followed by a 100 ms blank screen after which a red “X” appeared in the center of the screen that prompted the participants to indicate whether they had heard the signal in the first or the second interval by pressing the z-key on the keyboard with their left index finger or the m-key on the keyboard with their right index finger, respectively. The sound level of the signals was increased or decreased adaptively to the performance of the participant according to a transformed and weighted up/down rule (García-Pérez, 1998). This adaptive way of measuring is more efficient (fast while accurate) than other classic psychophysical methods used to determine the threshold (e.g., method of constants or method of limits), because most observations are obtained around the level of interest (e.g., the 80% detection level) on the psychometric curve (Levitt, 1971; Leek, 2001; Kingdom and Prins, 2010). Efficiency is very important when investigating effects of mood, because induced moods last for a relatively short time period, up to 20 min depending on the type of induction and the tasks performed (Isen et al., 1976; Frost and Green, 1982; Isen and Gorgoglione, 1983). The task duration fell within this period: The average duration of the threshold task after the mood-induction procedure was *M* = 3 min 35 s (*SD* = 29 s) for Experiment 1. We used a combination of the 1 up/2 down rule and a ratio of the stepsize down and stepsize up of 0.548, which has been shown to reliably converge to 80.35% correct performance (García-Pérez, 1998). Thus, the sound level of the tone went up one step (e.g., 3 dB) after one incorrect trial, but went down one step only after two consecutive correct trials, with the stepsize up being 1.82 times the size of the step down. The initial stepsize down was 15 dB, which changed to 5 dB after two reversal points (trials at which the sound level changed from going up to down or vice versa) and to 3 dB after 4 more reversal points. The sound levels of tones at the last 10 reversal points were averaged to calculate the threshold, or sound level needed for 80.35% performance. The e-prime script for the adaptive procedure was adapted from Hairston and Maldjian (2009).

#### Experiment Procedure

After reading and signing an informed consent, participants were guided to a quiet dimly lit individual test cubicle. They were instructed about the flow of the experiment and how to rate their mood on the affect grid. They practiced with correct earphone insertion and the experimenter verified whether external sounds were indeed attenuated. They were seated in a comfortable chair at 50 cm from the computer monitor, where further instructions were provided. After filling out the first affect grid the participants were instructed about the threshold task. It was explained that the signal would be presented equally often in each interval, and that an answer was required on all trials even though the signal might be difficult to hear on some trials. Participants were also encouraged to keep paying attention to the task in these cases. Instructions stressed accuracy and all responses were self-paced. In order to get used to the task, participants carried out eight practice trials that were equal to the trials of the threshold task except that the sound level of the signals was kept at 68 dB SPL and that participants received feedback about their accuracy after each trial. Following the practice trials and the baseline threshold task the second mood rating was obtained. Subsequently, the mood induction procedure started. Participants were asked to write as many details as possible of a mood-appropriate event on a piece of paper provided. To encourage vivid imagination, participants who chose to write about the given scenario were asked to answer six questions specifying the situation (e.g., What is the name of your friend? How does he/she look? What are your first thoughts at that moment? What do you tell your friend? What will be the consequences?). It was emphasized that after the procedure participants could put their notes in an envelope and that their notes would be treated confidentially. Five minutes after the start of the mood induction procedure the third affect grid appeared on the screen indicated by a soft warning tone. When this grid was filled out the mood-induction procedure continued for another 5 min. At the end of the procedure the fourth affect grid was completed after which the participants proceeded to the test threshold task. Upon completion of the fifth affect grid, participants were instructed to go back to baseline mood levels. Participants who went through the sad or the anxious mood induction procedure were given candy to alleviate their mood more easily. Subsequently, they filled out two more affects grids and additional questionnaires, which are not presented in this paper, except for a question concerning whether the thoughts used in the mood induction procedure were based on real or fictional events (two answer options). The final affect grid (referred to as sixth) was taken before participants were thanked, debriefed and paid. **Figure 1B** shows an overview of the experimental procedure.

### Results

All reported analyses were analyses of variance (ANOVA) or *t*-tests unless indicated otherwise. For all analyses a significance level of α = 0.05 was used.

#### Mood Induction Manipulation Check

**FIGURE 2:**
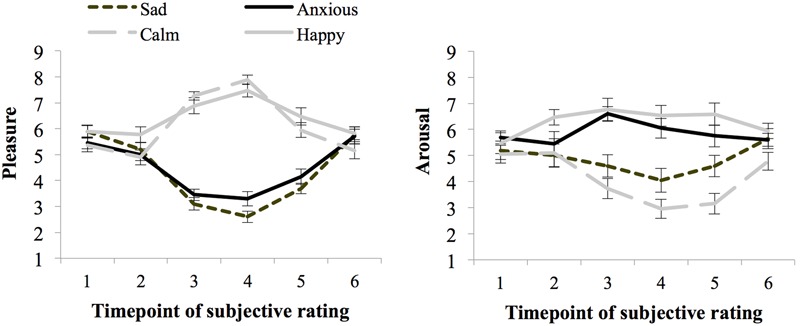
**Subjective ratings of pleasure and arousal levels during the experiment, per mood group (whiskers are standard errors) for Experiment 1**.

**Figure 2** shows the ratings of arousal and pleasure per moment of measurement during the experiment. Participants started out with a fairly neutral mood as reflected in the experienced level of arousal (*M* = 5.36 and *SE* = 0.17) and pleasure (*M* = 5.65 and *SE* = 0.13) at baseline [subjective rating 1 (SR1)]. There were no differences in subjective arousal or pleasure at baseline between the groups assigned to the moods (*p*s > 0.05).

Seventy-one percent of the participants indicated that they had used events that really happened for the writing and imagining task carried out during the mood induction procedure, and 29% indicated that they used fictional events. Subjective arousal and pleasure level averaged over ratings obtained before and after the threshold task (SR4 and SR5) indicate the experienced mood during task performance. The happy (*M* = 6.97 and *SE* = 0.24) and calm (*M* = 6.92 and *SE* = 0.23) groups experienced more pleasure than the anxious (*M* = 3.73 and *SE* = 0.23), and sad groups (*M* = 3.15 and *SE* = 0.22), *F*(1,72) = 240.67, *p* < 0.01, $\eta_{p}^{2}$ = 0.77, *MSE* = 1.00. Arousal ratings were higher for the high than the low-arousal groups, *F*(1,72) = 46.81, *p* < 0.01, $\eta_{p}^{2}$ = 0.39, *MSE* = 2.61. However, differences in arousal ratings between the happy and calm group were larger than between the anxious and sad group, as indicated by a significant interaction between pleasure and arousal, *F*(1,72) = 6.76, *p* = 0.011, $\eta_{p}^{2}$ = 0.09. Still, the Anxious group (*M* = 5.90, *SE* = 0.36) experienced more arousal than the sad group (*M* = 4.33, *SE* = 0.36), *F*(1,38) = 8.61, *p* < 0.01, $\eta_{p}^{2}$ = 0.19, *MSE* = 2.88. Similarly, the happy group (*M* = 6.56, *SE* = 0.32) experienced more arousal than participants in the calm group (*M* = 3.05, *SE* = 0.37), *F*(1,38) = 47.84, *p* < 0.01, $\eta_{p}^{2}$ = 0.59, *MSE* = 2.32.

#### Mood and Masked Threshold

**Table 2** shows the means and standard errors of the baseline and test thresholds for the different mood groups in dB SPL (for calculation of sound levels see method section). The baseline threshold did not differ between pleasure groups or between arousal groups, *F*s < 1, but there was an interaction between pleasure and arousal, *F*(1,72) = 4.90, *p* = 0.030, $\eta_{p}^{2}$ = 0.064, *MSE* = 3.53. However, independent *t*-test comparisons showed no significant differences between any of the four groups (all *p*s > 0.05). To reduce error variance, the baseline threshold was added as a covariate, *F*(1,71) = 3.57, *p* = 0.06, $\eta_{p}^{2}$ = 0.048, *MSE* = 2.69, in the analyses of the test threshold. The assumption of homogeneity of regression slopes was met, as indicated by the absence of an interaction between baseline threshold, arousal, and pleasure, *F*(3,68) = 1.70, *p* = 0.18 $\eta_{p}^{2}$ = 0.071, *MSE* = 2.61. Analysis of covariance (ANCOVA) showed that the threshold adjusted for the baseline threshold was higher in the high-arousal groups (adjusted *M* = 21.78 and *SE* = 0.27) than in the low-arousal groups (adjusted *M* = 20.76 and *SE* = 0.26), *F*(1,71) = 7.93, *p* = 0.008, $\eta_{p}^{2}$ = 0.094, *MSE* = 2.69. There was no effect of pleasure, *F* < 1, and the interaction effect between pleasure and arousal did not reach significance, *F*(1,71) = 2.89, *p* = 0.093, and $\eta_{p}^{2}$ = 0.039. The trend toward an interaction effect was due to a stronger effect of arousal in the high-pleasure moods, *F*(1,33) = 12.11, *p* = 0.001, $\eta_{p}^{2}$ = 0.27, *MSE* = 1.71 compared to the effect of arousal in the low-pleasure moods, *F* < 1, while the direction of these effects was the same in both the low- and high-pleasure moods.

**Table 2:** Baseline and test threshold (dB) per mood group of Experiment 1.

Threshold	Mood group			
	Low pleasure		High pleasure	
	Low arousal (Sad, *N* = 20)	High arousal (Anxious, *N* = 20)	Low arousal (Calm, *N* = 19)	High arousal (Happy, *N* = 17)
	*M* (*SE*)	*M* (*SE*)	*M* (*SE*)	*M* (*SE*)
Baseline threshold	20.66 (0.37)	21.71 (0.48)	21.39 (0.42)	20.53 (0.44)
Test threshold	20.99 (0.41)	21.56 (0.41)	20.50 (0.35)	22.02 (0.23)

Given the main effect of arousal and because the relation between arousal and task performance is often described by the classic inverted U-shaped Yerkes–Dodson curve (Easterbrook, 1959; Kahneman, 1973; Aston-Jones and Cohen, 2005), we performed a second-order polynomial sequential regression analysis of the masked-auditory threshold on subjective arousal during task performance centered to the mean, after first regressing out the baseline threshold. Because lower threshold indicates better task performance, we expected an upward U-shaped relation between subjective arousal and threshold.

**FIGURE 3:**
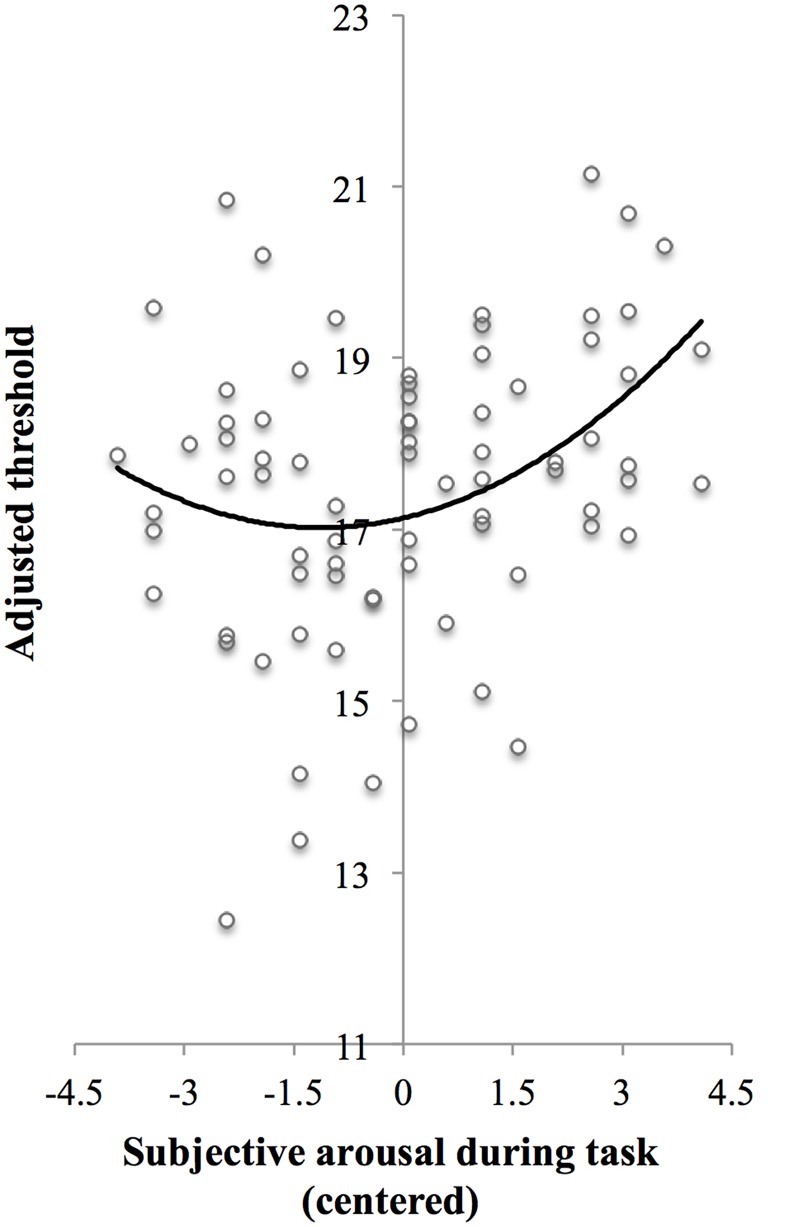
**Scatter plot of adjusted threshold versus centered subjective arousal for Experiment 1.** To be able to visualize the threshold as a function of arousal while controlling for baseline threshold, threshold scores were adjusted as follows: The threshold scores were fitted to a regression model *Y ′ = B_0_ + B_1_X_1_ + B_2_X_2_ + B_3_*$X_{2}^{2}$, where *Y′* is predicted test threshold, *X*_1_ is baseline threshold, and *X*_2_ is centered subjective arousal [regression coefficients (*B*) are presented in Step 3 in **Table 3**]. Next, the threshold scores (*Y*) were adjusted so that *Y _adjusted_ = Y-B_1_X_1_*. The solid curve shows the quadratic polynomial regression line of adjusted threshold on centered arousal scores, thus representing *Y*_adjusted_′ = *B*_0_ +*B*_2_*X*_2_ +*B*_3_*X*_2_^2^.

In line with the main effect of arousal found in the ANCOVA, adding centered subjective arousal to the regression model did improve prediction of the test threshold, $R_{\mathrm{change}}^{2}$ = 0.07, *F*_Change_(1,73) = 5.84, *p* = 0.018, compared to the model with the baseline threshold only, *R^2^* = 0.03, *F*(1,74) = 2.40, *p* = 0.13. Importantly, adding squared centered subjective arousal to the model with baseline threshold and centered subjective arousal further improved prediction of test threshold, $R_{\mathrm{change}}^{2}$ = 0.05, *F*_Change_(1,72) = 4.17, *p* = 0.045, which suggests the presence of a U-shaped relation between arousal and threshold in addition to the linear relationship. **Table 3** shows the beta values with standard errors and standardized betas per predictor. For the purpose of visualization, **Figure 3** shows a scatter plot of individual threshold scores adjusted for the baseline threshold scores as a function of centered subjective arousal scores and the quadratic polynomial regression line.

**Table 3:** Unstandardized regression coefficients (*B*), standardized regression coefficients (β), and *p*-values for the regression of test threshold on: baseline threshold (Step 1); baseline threshold and centered subjective arousal (Step 2); baseline threshold, centered subjective arousal, and squared-centered subjective arousal (Step 3) of Experiment 1.

	*B (SE)*	β	*p*
**Step 1**			
Intercept	17.86 (2.20)		<0.01
Base line threshold	0.16 (0.10)	0.18	0.13
**Step 2**			
Intercept	17.74 (2.13)		<0.01
Base line threshold	0.17 (0.10)	0.18	0.10
Linear-centered arousal	0.22 (0.090)	0.27	0.02
**Step 3**			
Intercept	17.13 (2.10)		<0.01
Base line threshold	0.18 (0.10)	0.20	0.08
Linear-centered arousal	0.2 (0.09)	0.24	0.03
Quadratic-centered arousal	0.09 (0.04)	0.22	0.05

### Discussion

The results of Experiment 1 suggest that affective arousal modulates basic auditory processing as measured by the masked-auditory detection threshold. The masked-auditory threshold was lower for people in a low-arousal mood (calm or sad), than for people in a high-arousal mood (happy or anxious). This suggests that affective arousal decreases masked sensitivity to pure tones. No effects of the pleasure level of the mood state were found.

These results may seem surprising given earlier demonstrations of augmented auditory-evoked responses in brief highly aroused affective states (Baas et al., 2006; Al-Abduljawad et al., 2008) and of increased loudness perception in negative high-arousal mood states (Siegel and Stefanucci, 2011). However, our findings may fit with cognitive and neuro-computational theories of performance and arousal. In his seminal work on affective arousal and performance, Easterbrook (1959) suggested that arousal narrows attention to task-relevant information. Up to a certain point this is beneficial for performance, but when relevant information falls outside the narrowing attentional focus, performance deteriorates (Easterbrook, 1959). This idea was complemented by Kahneman (1973) who proposed that in addition to a more narrow attentional focus, this focus is allocated in a more labile manner in high-arousal states. This also results in impaired performance at high-arousal levels due to increased distractibility. More recently, Aston-Jones and Cohen (2005) have proposed a neuro-computational mechanism for the relation between arousal and performance that links increasing arousal, including affective arousal (Aston-Jones et al., 1996), to the increase in tonic (baseline) NE release from the LC.

The LC is a nucleus in the brain stem and the brain’s main site of NE production. It modulates many brain areas through its extensive projections. Analogous to the inverted U-shaped relation between arousal and task performance (Easterbrook, 1959; Kahneman, 1973), animal research has shown that the level of tonic activity of the LC also relates to performance on target detection tasks according to an inverted U function (Aston-Jones et al., 1999; Usher et al., 1999; Aston-Jones and Cohen, 2005). With low levels of baseline (tonic) LC activity, behavior is characterized by inattentiveness and non-alertness. Increases in tonic LC level are associated with an improvement in performance. In this mode of intermediate baseline LC activity, also referred to as “phasic” mode, target stimuli, but not distractor stimuli, elicit strong phasic bursts of LC firing. This results in high levels of NE release in LC projection areas, where NE increases gain of target neurons (Berridge and Waterhouse, 2003), thereby increasing the signal to noise ratio (Servan-Schreiber et al., 1990). These phasic responses are associated with an increase in behavioral responsiveness to targets and thus an improvement in performance (Aston-Jones et al., 1999; Clayton et al., 2004; Aston-Jones and Cohen, 2005). When tonic LC activity further increases to high levels this is referred to as “tonic mode”. In this mode, there is hardly any discriminative phasic responding to target stimuli anymore, which is accompanied by a drop in target detection performance and behavior that is characterized by distractibility, labile attention focus, and scanning of the environment (Usher et al., 1999; Aston-Jones and Cohen, 2005). These findings from animal research are in line with recent observations in humans of increased distractibility by task-irrelevant stimuli on a visual pop-out distracter task in high-arousal moods (Kuhbandner and Zehetleitner, 2011). It is also in line with the older ideas of Kahneman (1973) on increased distractibility in high-arousal states.

Locus coeruleus baseline activity also directly influences responsiveness of sensory neurons. This has been demonstrated in a study in which tonic LC firing in rats was directly manipulated through electrical stimulation. Sensory-evoked responses of ensembles of somatosensory thalamus neurons were modulated by LC activity according to an inverted U-shape function (Devilbiss and Waterhouse, 2004). A more recent study also investigated changes in responsiveness of neurons in the auditory thalamus and auditory cortex to tones with concomitant phasic LC stimulation. This study showed that about half of the measured-evoked responses in thalamus and auditory cortex increased when accompanied by phasic LC stimulation compared to tone-only trials. This suggests that LC firing also modulates auditory responsiveness (Edeline et al., 2011).

Taken together, high-arousal affective states may be mediated by elevated tonic LC firing (Aston-Jones et al., 1996) and LC tonic firing mode decreases sensory (Devilbiss and Waterhouse, 2004) and behavioral responsiveness to targets (Aston-Jones et al., 1999; Aston-Jones and Cohen, 2005) which results in lower performance. This is in agreement with cognitive theories of arousal and performance (Kahneman, 1973). Our finding of increased masked-auditory threshold, indicating decreased ability for detecting target tones in high arousal compared to lower arousal states, may thus be explained by differences in tonic LC firing mode between these states. This idea is further supported by the curvilinear relationship we found between threshold and subjective arousal. Listeners who reported very low-subjective arousal or very high-subjective arousal had higher thresholds (lower performance) than listeners with a more intermediate level (higher performance).

Our results also seem to be in line with another recent account of the effects of arousal on perception, the arousal-biased competition (ABC) theory. According to this theory, arousal enhances the competition between stimuli competing for representation (Mather and Sutherland, 2011; Lee et al., 2012; Lee et al., 2014b). This results in heightened processing of salient stimuli at the cost of processing of non-salient stimuli. Furthermore, stimuli with similar salience that compete for representation mutually suppress each other’s activation. Because arousal enhances the competition, it decreases activation of the representations even further (Lee et al., 2012, 2014b). In the present study, the target stimuli (1000 Hz tones) were presented at threshold level and thus had similar salience to the competing background stimulus (masking noise), and activation of the representations for both stimuli would thus be suppressed. Following ABC theory, arousal further suppresses activity of the representations, resulting in need for higher salience of the target tone to be detected, and thus in a higher detection threshold.

As discussed above, the results of Experiment 1 suggest that irrespective of pleasure level, affective arousal impacted auditory masked sensitivity as measured by the masked-auditory threshold and this effect fits with findings on performance changes associated with changes in arousal and tonic NE levels and with the ABC theory. However limitations to Experiment 1 warrant caution in drawing a firm conclusion about the effect of mood on auditory masked sensitivity as measured by the masked-auditory threshold.

Although we took care to control for sound level of the musical pieces used for the mood induction, it was not possible to control for all other acoustic properties of the music, such as tempo, mode (minor and major) and other spectral properties. It is those properties that contribute to the differences in pleasure and arousal evoked by the different music pieces (Hunter et al., 2010). Because our dependent measure concerned performance on an auditory task, differences in acoustic properties may have directly influenced performance on this task and thus may have confounded the experiment. Indeed, effects of the frequency of a physical or imagined tone (cue) presented before each detection trial and expectancy of the target tone frequency during the whole task on masked sensitivity have been found (Tan et al., 2008; Borra et al., 2013). It should be noted, however, that these studies did not investigate effects of prior exposure to musical pieces on masked sensitivity.

To control for possible confounding by acoustic properties of the music, in Experiment 2, we carried out a study using an identical design to that of Experiment 1 with the exception that we used pictures to complement the mood induction procedure instead of music. If the finding of Experiment 1 that individuals in low (up to an optimal point) arousal mood had lower threshold than individuals in a high-arousal mood was a true effect of arousal, we expect similar findings for Experiment 2.

## Experiment 2: Masked-Auditory Threshold in Moods Induced by Pictures and Imagining

### Method

#### Participants

Power analysis in G^∗^Power 3 (Faul et al., 2007) indicated a desirable sample size of 78, using a power of 0.80, an effect size of *f* = 0.32 (equivalent to $\eta_{p}^{2}$ = 0.094, the arousal effect size in Experiment 1), an ANCOVA identical to Experiment 1, and α at 0.05. We recruited 84 female participants (Age: *M* = 19.5 and *SD* = 1.7, 17–24 years) with no self-reported hearing problems or depression to take part either for course credit or payment (€6.50). They were randomly assigned to one of four mood groups: calm, happy, sad, and anxious. Data from three participants were not included in the analyses because they had strongly deviating baseline or test thresholds (above the three inter quartile range criterion of the assigned mood group), and data from one participant could not be included because these were incomplete due to technical failure during data collection.

#### Materials

Apparatus, sound levels, mood assessment and threshold task were as described for Experiment 1, with the exception that foam ear tips were used for the insert earphones, providing 48 dB external noise attenuation.

#### Mood Induction and Assessment

The mood induction method was identical to the method used in Experiment 1 with the exception that imagining of the mood-appropriate event was combined with watching mood appropriate pictures instead of listening to music. The pictures were presented before the imagination task and consisted of 12 pictures per mood condition^1^ that were taken from the International Affective Picture System (IAPS, Lang et al., 2005). To create the sets of pictures for each of the four mood conditions, pictures were selected based on the average pleasure and arousal ratings [on a scale from 1 (low) to 9 (high)] for women as provided by the IAPS manual (Lang et al., 2005). This resulted in 1 set of 12 pictures depicting high-arousing unpleasant scenes (e.g., dangerous animals and crime scenes); 1 set of 12 pictures depicting low-arousing unpleasant scenes (e.g., funeral scenes and scenes depicting poverty, pollution, incarceration and people suffering from old age); 1 set of 12 pictures depicting high-arousing pleasant scenes (e.g., extreme sports scenes and romantic scenes); and 1 set of 12 pictures depicting low-arousing pleasant scenes (e.g., plants with flowers and peaceful nature scenes). **Table 4** shows the average ratings of the pictures per mood condition.

**Table 4:** Average ratings of the IAPS pictures used per mood condition in Experiment 2.

Mood condition	Mean pleasure rating (*SD*)	Mean arousal rating (*SD*)
Anxious (Low pleasure, High arousal)	2.30 (0.65)	6.89 (0.34)
Sad (Low pleasure Low arousal)	3.17 (0.58)	3.98 (0.21)
Happy (High pleasure, High arousal)	7.31 (0.64)	6.37 (0.52)
Calm (High pleasure, Low arousal)	6.89 (0.59)	3.16 (0.48)

*Ratings are taken from the normative ratings for women in the IAPS manual (Lang et al., 2005).*

#### Experiment Procedure

The only differences in procedure between Experiment 1 and Experiment 2 concerned the mood induction and the inclusion of female participants only. As in Experiment 1, the mood induction procedure started after the second mood rating was obtained. To help them to activate the desired mood state, participants first watched the 12 IAPS pictures, each presented for 5 s on the screen. Next they proceeded to the writing task, which was identical to the task in Experiment 1, however, in contrast to Experiment 1 there was no music playing in the background. Subsequently, they carried out the test threshold task, which had an average duration of *M* = 3 min 53 s (*SD* = 29 s).

### Results

All reported analyses were ANOVAs or *t*-tests unless indicated otherwise. For all analyses a significance level of α = 0.05 was used.

#### Mood Induction Manipulation Check

**FIGURE 4:**
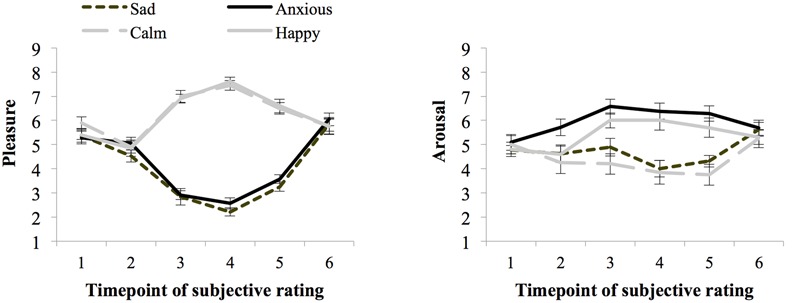
**Subjective ratings of pleasure and arousal levels during the experiment, per mood group (whiskers are standard errors) for Experiment 2**.

**Figure 4** shows the ratings of arousal and pleasure per moment of measurement during the experiment. Participants started out with a fairly neutral mood as reflected in the experienced level of arousal (*M* = 4.92 and *SE* = 0.16) and pleasure (*M* = 5.48 and *SE* = 0.12) at baseline (SR1). There were no differences in subjective arousal or pleasure at baseline between the groups assigned to the different moods (*p*s > 0.05).

Seventy-three percent of the participants indicated that they had used events that really happened for the writing and imagining task carried out during the mood induction procedure and 23% indicated that they used fictional events. During task performance (SR4 and SR5 averaged) the Happy (*M* = 7.10 and *SE* = 0.22) and Calm (*M* = 6.98 and *SE* = 0.17) groups experienced more pleasure than the Anxious (*M* = 3.07 and *SE* = 0.17), and Sad (*M* = 2.74 and *SE* = 0.15) groups, *F*(1,76) = 520.13, *p* < 0.01, $\eta_{p}^{2}$ = 0.87, *MSE* = 0.66. Arousal ratings were higher for the Happy (*M* = 5.85 and *SE* = 0.33) and Anxious (*M* = 6.33 and *SE* = 0.32) groups, than the Calm (*M* = 3.8 and *SE* = 0.33), and Sad (*M* = 4.16 and *SE* = 0.34) groups, *F*(1,76) = 40.59, *p* < 0.01, $\eta_{p}^{2}$ = 0.35, *MSE* = 2.20.

#### Mood and Masked Threshold

**Table 5** shows the means and standard errors of the baseline and test thresholds for the different mood groups in dB SPL. The baseline threshold did not differ between pleasure groups, *F* < 1, or between arousal groups, *F*(1,76) = 2.08, *p* = 0.15, $\eta_{p}^{2}$ = 0.027, *MSE* = 4.00, and there was no interaction between pleasure and arousal, *F*(1,76) = 1.99, *p* = 0.16, $\eta_{p}^{2}$ = 0.026. To reduce error variance, the baseline threshold was added as a covariate, *F*(1,75) = 15.63, *p* < 0.001, $\eta_{p}^{2}$ = 0.172, *MSE* = 0.81, in the analyses of the test threshold. The assumption of homogeneity-of-regression-slopes was met, as indicated by the absence of an interaction between baseline threshold, arousal, and pleasure, *F* < 1. The threshold adjusted for the baseline threshold was lower for the high-arousal groups (adjusted *M* = 16.23 and *SE* = 0.31) than for the low-arousal groups (adjusted *M* = 17.01 and *SE* = 0.31), but the ANCOVA showed that this effect did not reach significance, *F*(1,75) = 3.11, *p* = 0.082, $\eta_{p}^{2}$ = 0.040, *MSE* = 3.75. There was no effect of pleasure, or an interaction effect of pleasure and arousal, *F*s < 1.

**Table 5:** Baseline and test threshold (dB) per mood group of Experiment 2.

Threshold	Mood group			
	Low pleasure		High pleasure	
	Low arousal (Sad, *N* = 19)	High arousal (Anxious, *N* = 21)	Low arousal (Calm, *N* = 20)	High arousal (Happy, *N* = 20)
	*M* (*SE*)	*M* (*SE*)	*M* (*SE*)	*M* (*SE*)
Baseline threshold	16.39 (0.55)	16.37 (0.44)	17.22 (0.39)	15.94 (0.40)
Test threshold	17.06 (0.56)	15.86 (0.42)	17.25 (0.48)	16.33 (0.41)

To explore whether the relation between the threshold and subjective arousal is consistent with the inverted U-shaped relation between arousal and task performance curve (Easterbrook, 1959; Kahneman, 1973; Aston-Jones and Cohen, 2005), we performed a second-order polynomial sequential regression analysis of the masked-auditory threshold on subjective arousal during task performance centered to the mean, and after first regressing out the baseline threshold. The regression model including only the baseline threshold significantly predicted the test threshold, *R*^2^ = 0.18, *F*(1,78) = 17.56, *p* < 0.001. Improvement of prediction by adding centered subjective arousal did not reach significance, $R_{\mathrm{change}}^{2}$ = 0.033, *F*_Change_(1,77) = 3.22, *p* = 0.08, and further adding of squared centered subjective arousal did not improve prediction, $R_{\mathrm{change}}^{2}$ = 0.01, *F*_Change_(1,76) < 1. **Table 6** shows the beta values with standard errors and standardized betas per predictor.

**Table 6:** Unstandardized regression coefficients (*B*), standardized regression coefficients (β), and *p*-values for the regression of test threshold on: baseline threshold (Step 1); baseline threshold and centered subjective arousal (Step 2); baseline threshold, centered subjective arousal, and squared-centered subjective arousal (Step 3) of Experiment 2.

	*B* (*SE*)	β	*p*
**Step 1**			
Intercept	9.07 (1.81)		<0.01
Base line threshold	0.46 (0.11)	0.43	<0.01
**Step 2**			
Intercept	9.40 (1.80)		<0.01
Base line threshold	0.44 (0.11)	0.41	<0.01
Linear-centered arousal	-0.22 (0.12)	-0.18	0.08
**Step 3**			
Intercept	9.62 (1.82)		<0.01
Base line threshold	0.43 (0.11)	0.41	0
Linear-centered arousal	-0.23 (0.12)	-0.19	0.06
Quadratic-centered arousal	-0.05 (0.07)	-0.08	0.45

### Combined Results of Experiment 1 and Experiment 2

To explore whether or not the results of Experiment 2 were different from the results of Experiment 1, we combined the data from both experiments and examined if there were any interactions with experiment. Because the values of the (baseline) thresholds differed between Experiment 1 and Experiment 2 (possibly due to use of different material, e.g., ear tips), we used normalized scores. Baseline and test threshold scores were normalized to the baseline threshold of the respective experiment. This was done in the following way: The experiment mean of the baseline threshold was subtracted from the individual test threshold scores and these differences were divided by the experiment standard deviation of the baseline threshold.

#### Mood Induction Manipulation Check

To check whether the effect of the mood induction affected experienced arousal and pleasure mood differently for both experiments interactions between mood group and experiment on arousal and pleasure ratings (averaged over SR4 and SR5) were examined.

In addition to a main effect of pleasure, *F*(1,148) = 702.55, *p* < 0.001, $\eta_{p}^{2}$ = 0.83, *MSE* = 0.808 on pleasure experienced during task performance, there was a significant interaction effect between experiment and pleasure, *F*(1,148) = 4.70, *p* = 0.03, $\eta_{p}^{2}$ = 0.31, *MSE* = 0.808. The interaction effect occurred due to lower pleasure ratings in the low-pleasure moods in Experiment 2 (*M* = 2.9, *SE* = 0.14) than in Experiment 1 (*M* = 3.44, *SE* = 0.14), *F*(1,148) = 7.13, *p* = 0.009, $\eta_{p}^{2}$ = 0.83, *MSE* = 0.808. There were no differences between experiments for pleasure ratings in the high-pleasure moods, *F* < 1. As can be seen from the analyses presented above for each experiment separately, the differences between low and high-pleasure moods were large and significant for both experiments.

**FIGURE 5:**
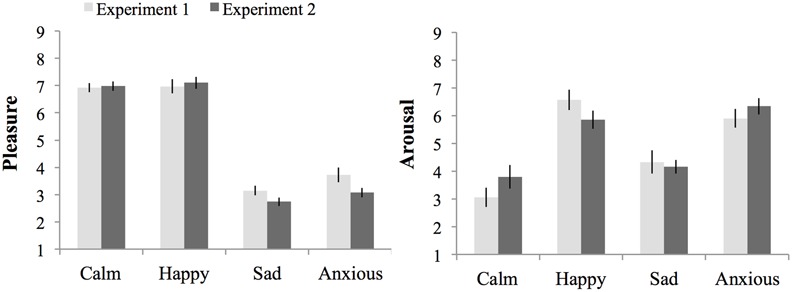
**Subjective pleasure and subjective arousal during threshold task performance per mood condition per experiment.** Errors bars represent 1 SE above and below the means.

In addition to the main effect of arousal, *F*(1,148) = 210.38, *p* < 0.001, $\eta_{p}^{2}$ = 0.37, *MSE* = 2.40, on arousal experienced during task performance there was a significant interaction between experiment, pleasure and arousal, *F*(1,148) = 4.29, *p* = 0.04, $\eta_{p}^{2}$ = 0.03, *MSE* = 2.40. This interaction effect was due to the presence of an interaction between pleasure and arousal in Experiment 1 that occurred because the effect of arousal was larger in the high-pleasure than low-pleasure moods, which was absent in Experiment 2 (see Results for each experiment separate above). There was no main effect of experiment or an interaction effect between experiment and arousal, *F*s < 1. **Figure 5** shows the subjective pleasure and arousal during threshold task performance per mood condition per experiment.

#### Mood and Masked Threshold

**FIGURE 6:**
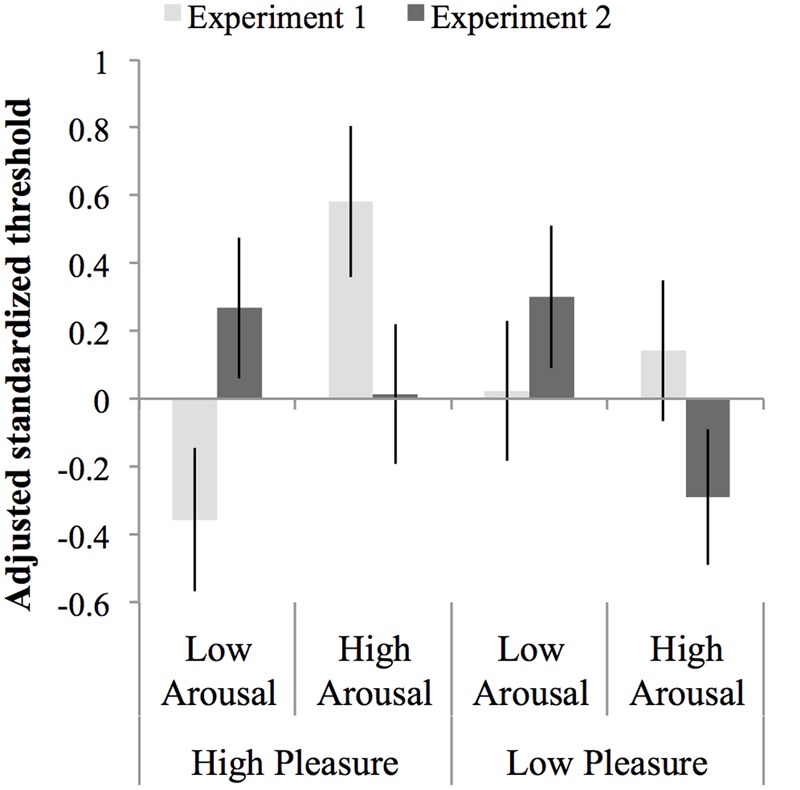
**Thresholds normalized to the mean and standard deviation of the baseline threshold and adjusted for the baseline threshold per experiment per mood condition.** Errors bars represent 1 SE above and below the mean.

An ANCOVA was carried out on the normalized test threshold, with standardized baseline threshold as covariate, *F*(1,147) = 17.68, *p* < 0.001, $\eta_{p}^{2}$ = 0.107, *MSE* = 0.84 and experiment, arousal and pleasure as factors. The assumption of homogeneity of regression slopes was met, as indicated by the absence of an interaction between baseline threshold, experiment, arousal, and pleasure, *F*(7,140) = 1.31, *p* = 0.25, $\eta_{p}^{2}$ = 0.062, *MSE* = 0.83. The ANCOVA showed no main effect of arousal, pleasure or experiment, *F*s < 1, but did show an interaction between arousal and experiment, *F*(1,147) = 10.33, *p* = 0.002, $\eta_{p}^{2}$ = 0.066, *MSE* = 0.84. This interaction occurred due to opposite effects of arousal in Experiment 1 compared to Experiment 2: in Experiment 1 thresholds were larger in the high-arousal than the low-arousal groups, while in Experiment 2 thresholds were smaller, albeit not significantly, in the high than in the low-arousal groups (see Section “Results” for each Experiment separate above). There were no other interactions with experiment, *F* < 1, but the interaction between pleasure and arousal approached significance, *F*(1,147) = 3.71, *p* = 0.056, $\eta_{p}^{2}$ = 0.107, and *MSE* = 0.03. This interaction occurred because the effect of arousal in the low-pleasure groups was opposite to the effect of arousal in the high-pleasure groups, however, both effects of arousal were not significant, *F*(1,75) = 1.22, *p* = 0.27, $\eta_{p}^{2}$ = 0.02, *MSE* = 0.98, and *F*(1,75) = 2.42, *p* = 0.12, $\eta_{p}^{2}$ = 0.12, *MSE* = 0.70, respectively. **Figure 6** shows an overview of the normalized thresholds adjusted for the standardized baseline threshold per experiment per mood condition.

To explore whether the combined data of Experiment 1 and 2 are consistent with the inverted U-shaped relation between arousal and task performance curve (Easterbrook, 1959; Kahneman, 1973; Aston-Jones and Cohen, 2005), we performed a second-order polynomial sequential regression analysis of the normalized masked-auditory threshold on subjective arousal during task performance centered to the mean (of Experiment 1 and 2 together), and after regressing out the standardized baseline threshold. The regression model including only the baseline threshold significantly predicted the test threshold, *R*^2^ = 0.10, *F*(1,154) = 17.02, *p* < 0.001. Adding centered subjective arousal did not improve prediction of the model, $R_{\mathrm{change}}^{2}$ = 0.001, *F*_Change_(1,153) < 1 and neither did further adding of squared centered subjective arousal, $R_{\mathrm{change}}^{2}$ = 0.01, *F*_Change_(1,152) < 1. **Table 7** shows the beta values with standard errors and standardized betas per predictor.

**Table 7:** Unstandardized regression coefficients (*B*), standardized regression coefficients (β), and *p*-values for the regression of test threshold (normalized to the mean and standard deviation of the baseline threshold) on: baseline threshold (Step 1); baseline threshold and centered subjective arousal (Step 2); baseline threshold, centered subjective arousal, and squared-centered subjective arousal (Step 3).

	*B* (*SE*)	β	*p*
**Step 1**			
Intercept	0.08 (0.08)		0.324
Base line threshold	0.31 (0.08)	0.32	<0.01
**Step 2**			
Intercept	0.08 (0.08)		0.325
Base line threshold	0.32 (0.08)	0.32	<0.01
Linear centered arousal	0.02 (0.04)	0.03	0.703
**Step 3**			
Intercept	-0.02 (0.11)		0.875
Base line threshold	0.32 (0.08)	0.32	<0.01
Linear-centered arousal	0.02 (0.04)	0.03	0.697
Quadratic-centered arousal	0.02 (0.02)	0.09	0.223

*Data of Experiment 1 and 2 were combined for this analysis.*

## General Discussion

We investigated the effect of the pleasure and arousal dimension of mood on the masked-auditory threshold in two experiments. In Experiment 1, the mood induction procedure was accompanied by music, while in Experiment 2 a visual mood induction procedure was used. Experiment 1 suggested that lower (up to a certain optimum) affective arousal decreased the masked-auditory threshold, irrespective of pleasure level. However, as indicated by an interaction effect between experiment and arousal, arousal did not have the same effect in Experiment 2. The effect of arousal in Experiment 2 on the masked-auditory threshold did not reach significance, but was a trend in the opposite direction to the effect in Experiment 1. In both experiments, no significant effects of the pleasure level were found on the masked-auditory threshold. The remainder of the discussion will, therefore, focus on the effects of the arousal dimension rather than the pleasure dimension of mood on auditory processing.

Although we carefully controlled the music for sound level in Experiment 1, Experiment 2 was carried out to exclude any possibility of confounding by other acoustic properties of the music used for the mood induction. To this end affective pictures instead of music were used for the mood induction procedure in Experiment 2. Analysis of self-reported arousal and pleasure experienced during the threshold task after the mood induction showed that the participants in both experiments had the desired mood states during the task. Therefore, if the effects on the threshold found in Experiment 1 were due to differences in arousal, we would also expect these effects in Experiment 2. However, as noted above, arousal had a different effect in Experiment 2; there was a trend in opposite direction to the effect in Experiment 1.

We checked if the finding of opposite directions of the arousal–threshold relation in the two experiments could be explained by a curvilinear relation between arousal and threshold. The presence of a curvilinear relationship was expected based on theories about the relation between arousal and performance in general (Kahneman, 1973; Aston-Jones and Cohen, 2005), and the findings of Experiment 1. The results of Experiment 1 suggested a curvilinear relation between arousal and threshold, which reflected that listeners who reported very low subjective arousal or very high subjective arousal had higher thresholds (lower masked sensitivity) than listeners with a more intermediate (optimal) level. A curvilinear relation could explain opposite effects of arousal on the threshold in the following way: If on average the subjective arousal levels for participants in Experiment 1 fell on the higher side of the optimum, thus on the right side of the U curve, this would be reflected in a positive relation between arousal and threshold. And, if on average the subjective arousal levels for participants in Experiment 2 fell on the lower side of the optimum, thus on the left side of the U curve, this would be reflected in a negative relation between arousal and threshold. This, however, does not seem to be the case. The comparison of subjective arousal scores between the experiments did not reveal a statistically significant main effect of experiment, which suggests that average arousal scores did not differ between experiments. Furthermore, the regression analysis of the threshold for both studies together showed that squared arousal did not significantly improve prediction of the threshold beyond that of the baseline threshold (and linear arousal). Thus, given the distribution of the subjective arousal scores of both experiments and the analyses of the combined experiments, there is no basis to conclude that a U-shaped relation between arousal and the threshold could explain why Experiment 2 showed a trend in opposite direction to the effect of arousal Experiment 1.

Taken together, although in both experiments the different mood induction procedures had similar effects on subjectively experienced arousal, the effect of arousal on the auditory threshold differed per experiment, as indicated by the interaction effect between study and arousal. Therefore, it cannot be concluded that mood induction by music had the same effect as induction using pictures. Instead, the effect in Experiment 1 may have been brought about by differences in the acoustical properties of the music between mood conditions. Studies that systematically investigate the effects of acoustical properties of preceding auditory stimulation on subsequently measured thresholds could shed more light on this possibility.

Even though the mood induction was successful in both experiments, the inconsistent results between experiments may have also been caused by a larger difference in subjectively experienced arousal between calm and happy conditions in Experiment 1 compared to Experiment 2 (**Figure 5**). These numerical differences receive some support from an extra ANOVA of subjective arousal during the threshold in the high-pleasure conditions showing that the interaction between arousal and experiment almost reached significance: *F*(1,76) = 3.54 and *p* = 0.054. Also note that a separate ANOVA showed no significant interaction effect between arousal and experiment, *F* < 1, in low-pleasure conditions. The difference in successfulness of mood induction between experiments may, at least partly, explain the presence of an effect of arousal on the masked-auditory threshold in Experiment 1 and a weaker arousal effect (trend in opposite direction) in Experiment 2. However, it seems unlikely to fully explain the differences between the findings of Experiment 1 and 2. Furthermore, more complex interactions between mood and other factors associated with differences between the studies may also have occurred. For example, in Experiment 1, due to the use of music, attention may have been focused more on the auditory than the visual modality compared to Experiment 2. This, in turn, may have rendered the masked-auditory threshold task differently susceptible to mood effects compared to Experiment 2.

As set out above, in Experiment 1 the threshold significantly differed between the high- and low-arousal conditions. However, this difference may be explained by other factors than arousal *per se*. While these factors were excluded in Experiment 2, the results of Experiment 2 do not provide conclusive evidence regarding the presence of an arousal effect. Only a marginally significant effect was found. Furthermore, the number of participants for Experiment 2 was determined by a pre-study power analysis based on an effect of similar magnitude as the effect in Experiment 1. A direct replication of Experiment 2 using a much larger number of participants would be necessary to evaluate whether any effects, including small effects, of arousal on the masked-auditory threshold are present or not (Brandt et al., 2014; Simonsohn, 2015). In addition to large scale replication of the present study we would like to present additional suggestions for further research into the under-explored topic of affective modulation of basic auditory perception.

### Suggestions for Further Research

First, future studies could explore the effects of brief affective stimuli on the masked-auditory threshold. Mood by nature is long in duration; however, the effects of a mood induction may wane over time, which limits the duration of the auditory task that can be employed. Brief affective stimuli, such as affective pictures, presented before auditory task stimuli allow researchers to circumvent this limitation. In addition, using brief affect inductions allows for within subject comparison within one session.

Second, future studies may investigate the effects of more extreme affect inductions on the masked-auditory threshold instead of mood induction. Although subjective pleasure and arousal ratings showed that our mood manipulation changed people’s affective state successfully, mood states are more diffuse and less extreme than other types of affective state, such as the state elicited by the threat of shock paradigm. The latter could therefore be more effective in eliciting changes in early perception. Indeed, studies using this more extreme affect method found modulation of a very early stage of auditory processing in the brain (Baas et al., 2006).

Third, future studies may include parametric manipulation of arousal with both extreme and more intermediate arousal conditions. As discussed above, Experiment 1 showed a curvilinear relationship between subjectively experience arousal and the masked-auditory threshold. However, a similar pattern was not found in Experiment 2, and the relationship in Experiment 1 was based on subjectively experienced rather than experimentally controlled arousal levels. In order to provide more definite conclusions regarding non-linear effects of arousal, for future research it is advisable to include both extreme and intermediate arousal conditions.

Fourth, future studies should employ various tasks tapping into different aspects of early auditory perception. Previous studies showed that improvement of early visual perceptual processing by affect depends on the properties of the stimuli that are processed. For example, Bocanegra and Zeelenberg (2009) and Lee et al. (2014a) demonstrated that brief presentation of fear-inducing stimuli enhanced subsequent processing of low-spatial-frequency visual stimuli, while it impaired processing of high-spatial frequency visual stimuli. Similarly, modulation of sensitivity of auditory perception is likely to be dependent on the type of stimuli employed. The present study explored effects on the masked-auditory threshold for 1 kHz tones using simultaneous energetic masking conditions. Future studies into affective modulation of early auditory perception should also explore effects of affective arousal on various other tasks and stimuli. For example, effects of arousal on simultaneous masking may be compared to effects on backward masking. In a backward-masking task, the mask is presented directly after the detected tone. This task taps into temporal auditory processing and is thought to be more susceptible to cognitive modulation (Strait et al., 2010) and may therefore also be more susceptible to modulation by affective arousal.

Fifth, future research may simultaneously measure affect modulation of bias and sensitivity (in terms of signal detection theory). As set out in the introduction, previous findings of increased perceived loudness in high-arousal negative mood (Siegel and Stefanucci, 2011) could be brought about by increased auditory sensitivity and by mood effects on the criterion for responding and bias judgments. Therefore, in the current study, we took care to exclude effects of bias by using a 2IFC task, which is designed to provide a measure of perceptual sensitivity and control for bias (Green and Swets, 1966). This task was chosen for its relative time efficiency in order to stay within the duration of the induced mood. However, the task does not allow for statements about the relative contributions of bias and sensitivity to mood modulation of auditory perception. Future studies could measure influences of affective arousal on the masked-auditory threshold by means of a signal detection task that provides separate indices of bias and sensitivity.

## Conclusion

Research into affective modulation of auditory processing is still in its infancy. Our study contributed to this field by investigating the effect of mood state on the masked-auditory detection threshold, a presumably criterion free measure of auditory-masked sensitivity. Our results showed no significant effect of pleasure level on auditory-masked sensitivity. The effect of the arousal level depended on the modality of the stimuli (auditory or visual) used in the mood induction, which makes it difficult to draw conclusions regarding the question whether the effect of arousal on the threshold is a genuine effect of mood. Future studies should investigate affective modulation of different aspects of audition using different types of affect modulations to elucidate which aspects of auditory processing are susceptible to modulation by affect and which are not.

## Ethics Statement

This study was approved by the ethics committee of the Institute of Psychology of Leiden University. All participants gave written informed consent prior to participation.

## Author Contributions

All authors were involved in designing the study. AB performed the research. AB and GB analyzed the data. AB drafted the manuscript with input from GB and PS. All authors critically revised the manuscript and approved the final version for submission.

## Conflict of Interest Statement

The authors declare that the research was conducted in the absence of any commercial or financial relationships that could be construed as a potential conflict of interest.

## Footnotes

1 IAPS pictures used. Anxious condition: 1120, 1200, 1300, 3071, 3530, 6230, 6370, 6510, 6540, 6570, 6821, 9252; Sad condition: 2490, 2590, 2722, 6010, 9001, 9045, 9090, 9101, 9110, 9190, 9220, 9331; Happy condition: 1650, 4611,5621, 5626, 5629, 8030, 8034, 8080, 8200, 8370, 8470, 8501; Calm condition: 1450, 1610, 1900, 2560, 5030, 5201, 5250, 5593, 5720, 5750, 5800, 7900.
